# Importance of Fc-mediated functions of anti-HIV-1 broadly neutralizing antibodies

**DOI:** 10.1186/s12977-018-0438-x

**Published:** 2018-08-22

**Authors:** Matthew S. Parsons, Amy W. Chung, Stephen J. Kent

**Affiliations:** 1 Department of Microbiology and Immunology, The University of Melbourne, at the Peter Doherty Institute for Infection and Immunity, Victoria, Australia; 2 ARC Centre of Excellence in Convergent Bio-Nano Science and Technology, The University of Melbourne, Victoria, Australia; 3 Melbourne Sexual Health Centre, Alfred Hospital, Monash University Central Clinical School, Victoria, Australia

**Keywords:** HIV-1, ADCC, Broadly neutralizing antibodies

## Abstract

Anti-HIV-1 broadly neutralizing antibodies (BnAbs) exhibit an impressive capacity to protect against chimeric SIV-HIV (SHIV) challenges in macaques and potently reduce viremia in both SHIV-infected macaques and HIV-1-infected humans. There is a body of evidence suggesting Fc-mediated functions of anti-HIV-1 binding antibodies are important in protecting from infection and controlling viremia. The degree to which the efficacy of BnAbs is assisted by Fc-mediated functions is of great interest. Challenge experiments with the older generation BnAb b12 showed that mutating the Fc region to abrogate Fcγ receptor binding reduced protective efficacy in macaques. Similar data have been generated with newer BnAbs using murine models of HIV-1. In addition, the degree to which therapeutically administered BnAbs reduce viremia suggests that elimination of infected cells through Fc-mediated functions may contribute to their efficacy. Fc-mediated functions that eliminate infected cells may be particularly important for challenge systems involving cell-associated virus. Herein we review data regarding the importance of Fc-mediated functions of BnAbs in mediating protective immunity and control of viremia.

## Introduction

An HIV-1 vaccine is urgently needed, and new technologies to control HIV-1 infection in the absence of lifelong antiretroviral drug therapy are being actively pursued. Many highly potent neutralizing antibodies that neutralize broad arrays of HIV-1 isolates, termed broadly neutralizing antibodies (BnAbs), have been isolated in recent years [1]. Passive transfer of these antibodies reliably protects macaques from exposure to cell free chimeric Simian-Human Immunodeficiency Virus (SHIV) and reduces viremia in SHIV-infected macaques and HIV-1-infected humans [2–12]. Passive transfer of the BnAb VRC01 is currently under evaluation for its potential to protect humans from HIV-1 (NCT02716675 and NCT02568215).

Coincident with this exciting work on BnAbs, there is a growing body of literature on the importance of Fc-mediated functions of HIV-1 antibodies. Fc-mediated functions of non-neutralizing antibodies appeared to be important in the modest protective efficacy of the RV144 HIV-1 vaccine regimen [13–15]. Fc-mediated functions of HIV-1 antibodies generally correlate with slow HIV-1 disease progression and can force viral escape [16–19].

More potent Fc-mediated functions of BnAbs should theoretically enhance their efficacy and there is some evidence that this is the case [20]. This might be particularly important when HIV-1 is transmitted in the context of infected cells, which may partially evade neutralization by BnAbs. This review summarizes data on the importance of Fc-mediated functions of BnAbs.

## Diversity of Fc-mediated functionality of isolated BnAbs

The breadth of viral recognition and much of the anti-viral potency of BnAbs is derived from the recognition of key viral epitopes by BnAb paratopes that prevent the infection of cellular targets through viral neutralization. Importantly, BnAbs have the potential to mediate a diverse array of additional non-neutralizing functions through ligation of the Fc portion of the antigen-bound antibody by components of the complement system or effector cells expressing Fc receptors (FcR). Indeed, HIV-1-infected cells bound by BnAbs can be targeted by FcR-expressing effector cells, such as natural killer (NK) cells, for elimination by antibody-dependent cellular cytotoxicity (ADCC) [21–23]. As well as cytolysis of infected cells opsonized by BnAbs, effector cells recognizing BnAb-coated target cells can become stimulated to produce soluble factors, such as beta chemokines, that can inhibit viral spread. The combination of ADCC, neutralization and effector cell derived soluble inhibitors of viral spread has been termed antibody-dependent cell-mediated viral inhibition (ADCVI), and this response can be mediated by BnAbs [24, 25]. Additionally, FcR-expressing phagocytic effector cells, such as monocytes, can eliminate BnAb-coated virions through an antibody-dependent uptake process, termed antibody-dependent phagocytosis (ADP) [26]. Lastly, infected cells coated by BnAbs can be targeted for elimination by the process of antibody-dependent complement-mediated lysis (ADCML) [22]. It should be noted that further diversity in these processes is introduced by the differential responsiveness of effector cells at different stages of ontogeny and differentiation, as well as polymorphisms in FcR that adjust effector cell responsiveness to antibody-coated target cells. Lastly, diversity in Fc-dependent non-neutralizing functions is driven by the differential capacity of individual BnAbs to trigger these functions.

Much research into the Fc-dependent functions of BnAbs has focused on ADCC. Indeed, several independent studies have assessed the capacity of panels of antibodies (including BnAbs) to trigger NK cell-mediated ADCC of target cells infected with diverse viral isolates [21–23]. Although these studies have revealed that the observed ADCC is highly dependent on the antibody and virus combination studied, several general characteristics of ADCC have been elucidated. It has now been demonstrated that: (1) the degree of antibody binding to target cells correlates with the susceptibility of the target cell to ADCC [21–23]; (2) the ability of the antibody to neutralize a virus isolate associates with the capacity of the antibody to trigger ADCC of target cells infected with the same isolate [21, 23]; and (3) combinations of antibodies trigger potent ADCC [21, 22]. It should be noted that exceptions to these generalizations have been reported. In particular, it has been reported that the 2G12 and 2F5 BnAbs trigger poor ADCC despite binding to infected cells [23]. Although the reasons for the reduced ADCC function of these two BnAbs have not been determined, roles for NK cell accessibility to 2F5 and the Fab swapped variable region of 2G12 have been proposed [23]. Importantly, some investigators have observed ADCC by 2F5 recognizing infected target cells [27], and 2G12 in monomeric and dimeric formats has been reported to induce ADCC [28, 29]. This suggests that the ADCC capacity of some BnAbs is highly context dependent, and this is an area for future research.

In addition to ADCC, recent research has also demonstrated BnAbs to bind to FcRs involved in ADP and to have the capacity to trigger phagocytosis of viral particles. Factors influencing BnAb ADP were recently investigated by Tay et al. [26]. These investigators employed the CD4 binding site specific CH31 BnAb to determine the effect of antibody isotype and IgG subclass on phagocytic activity. Primary monocytes were employed as effector cells and demonstrated to uptake viral particles opsonized by IgG1, monomeric IgA1 and monomeric IgA2 versions of CH31. Interestingly, IgG1 was a more potent inducer of viral particle uptake than IgA1 or IgA2. Next, the relative capacities of IgG1 and IgG3 to trigger ADP of viral particles by primary monocytes were assessed. The IgG3 version of CH31 was a more potent inducer of ADP than IgG1. This phenomenon of more potent ADP by IgG3 was shown to not be a result of enhanced antigen binding, and enhanced ADP of IgG3 compared to IgG1 was demonstrated for two additional BnAbs (CH27 and CH28) and non-broadly neutralizing antibodies directed to different epitopes.

As well as FcR binding, several BnAbs have now been screened for their capacity to trigger lysis of infected cells in a complement-dependent manner [22]. Furthermore, mutants of a CD4 binding site BnAb, b12, have been generated to exhibit different patterns of complement binding [30]. Passive immunization of these b12 mutants in macaques prior to mucosal SHIV challenge revealed no role for complement in b12-conferred protection from infection [5]. It remains undetermined if this observation extends to BnAbs other than b12. The capacity of a panel of antibodies (including BnAbs) to trigger ADCML of HIV-1-infected target cells was recently investigated by Mujib et al. [22]. An array of ADCML capacities was noted within the antibody panel. Although not statistically significant, a trend was noted between the level of antibody binding to infected cells and the ADCML observed.

As well as the Fc-dependent functions of BnAbs reviewed above, it is important to highlight additional functional roles of the antibody Fc that might be of significance for BnAb-conferred protection from HIV-1. For instance, interaction of antibody Fc with the neonatal FcR (FcRn) is important for extending antibody half-life, as well as localizing and sustaining antibody to mucosal sites of HIV-1 exposure [31]. Lastly, an understudied Fc-FcR interaction is that between antibodies and the inhibitory FcγRIIb. Interaction of immune complexes formed by live-attenuated SIV vaccine-induced antibodies with FcγRIIb expressed in the epithelium associates with live-attenuated vaccine-conferred protection from infection [32]. It is thought that this interaction prevents/diminishes the recruitment of target cells for SIV infection to the site of exposure. The importance of FcγRIIb for BnAb conferred protection from mucosal viral challenges in macaques has not yet been investigated.

## Role of Fc-dependent functions in efficacy of BnAbs against cell free virus in vivo

A collection of studies demonstrate that BnAbs administered passively or through gene transfer using adeno-associated viral vectors protect against in vivo challenge with cell free virus in animal models of HIV-1 infection [5–8, 10, 12, 20, 31, 33–36]. While these studies clearly highlight the prophylactic capacity of BnAbs, only a few of the reports incorporated experiments to assess the potential mechanisms of BnAb-conferred protection (Table 1) [5, 20, 31, 35, 36]. Such experiments are important to gauge if neutralization function is sufficient, or if BnAbs need to trigger additional non-neutralizing functions through their Fc portions to protect against HIV-1 exposure.

**Table 1:** Role for Fc-dependent BnAb functions for protection from cell free virus in vivo

Aim of study	BnAb studied	Model	Outcome	References
Compare wild type b12 with b12 versions deficient for FcR binding and/or complement binding for protection of macaques from high-dose SHIV challenge	b12	Macaque high-dose SHIV	Elimination of the ability of b12 to engage FcR diminished the ability of the antibody to protect macaques from high-dose SHIV challenge	[5]
Compare low doses of wild type b12 and b12 deficient for FcR binding for protection of macaques from repeated low-dose SHIV challenge	b12	Macaque repeated low-dose SHIV	More challenges did not result in infection of animals infused with wild type b12, as compared to animals infused with b12 deficient in FcR binding	[35]
Assess if low doses of a non-fucosylated version of b12, with enhanced ADCC potential, are better than wild type b12 for protecting macaques from repeated low-dose SHIV challenge	b12	Macaque repeated low-dose SHIV	Non-fucosylated b12 did not provide enhanced protection from repeated low-dose SHIV challenge, as compared to wild type b12	[36]
Screen panel of BnAbs with enhanced of diminished FcR binding for ability to block viral entry in a murine model	BnAb panel	Murine HIV-1 entry	BnAbs with enhanced FcR binding demonstrated enhanced in vivo blocking of HIV-1 entry	[20]
Determine if modifying VRC01 to enhance binding to FcRn improves the ability of suboptimal doses of the BnAb to protect against SHIV challenge	VRC01	Macaque SHIV	Suboptimal doses of VRC01 with enhanced binding to FcRn protected more macaques from SHIV challenge than wild type VRC01	[31]

Hessell et al. initially evaluated the involvement of non-neutralizing Fc-dependent functions in BnAb conferred protection from in vivo cell free virus challenge [5]. For this purpose the authors utilized a previously developed panel of variants of the CD4 binding site BnAb, b12 [30]. These variants included the b12 wild type (b12 WT), a version mutated to diminish FcγR and complement binding (b12 LALA), and a version only exhibiting diminished complement binding (b12 KA). Each of these b12 variants, or human IgG1 isotype control, were passively administered intravenously to rhesus macaques prior to vaginal challenge with high-dose cell free SHIV_SF162P3_. While all four animals receiving the isotype control were infected following challenge, animals receiving b12 WT and b12 KA exhibited similar robust levels of protection from infection (i.e. 8/9 animals protected in each group). Animals receiving the b12 LALA were less likely to be protected from challenge than animals receiving the b12 WT or b12 KA antibodies (i.e. 5/9 animals protected). These results imply that neutralization is often sufficient for protection from cell free virus challenge, but protection is optimized if BnAbs can trigger additional non-neutralizing effector cell functions through effector cells expressing FcγRs.

Following their observation of lower efficacy of b12 LALA than b12 WT in protecting against high-dose cell free virus challenge, Hessell et al. evaluated the potential role of non-neutralizing functions of b12 for protection of macaques from repeated low-dose cell free SHIV_SF162P3_ challenge [35]. Animals were injected once weekly with low doses of b12 WT or b12 LALA and challenged twice weekly with low-dose virus. Animals inoculated with either b12 WT or b12 LALA required more challenges prior to becoming infected than animals administered isotype control prior to challenge. Although both b12 WT and b12 LALA provided protection compared to isotype control, protection conferred by b12 LALA appeared suboptimal as compared to b12 WT. Indeed, nearly twice as many challenges did not result in infection for b12 WT animals than b12 LALA animals (i.e. 104 vs. 61). These observations largely reflected the relative patterns of protection conferred by b12 WT and b12 LALA following high-dose viral challenge [5].

The major implication of the two studies assessing the relative protective efficacy of b12 variants with different FcγR binding potential is that neutralization is often sufficient for protection from infection with cell free virus. It is possible that neutralization might fail to be sufficient for protection when too large of a number of cells are infected following challenge. In these situations non-neutralizing Fc-dependent functions of BnAbs could be required to purge infected cells through ADCC or eliminate the virions produced by infected cells by ADP. The potential contribution of ADCC to protection conferred by b12 was evaluated by Moldt et al., who compared the relative protection from repeated low-dose SHIV_SF162P3_ challenge conferred by b12 WT to a non-fucosylated version of b12 (NFb12) [36]. Despite exhibiting higher binding to human and rhesus macaque FcγRIIIa and mediating higher ADCC of HIV-1-infected cells, the NFb12 antibody did not confer enhanced protection from cell free viral challenge as compared to b12 WT. These results might reflect the importance of Fc-dependent functions other than ADCC in the protection conferred by BnAbs.

Until recently much of the research into the role of non-neutralizing Fc-dependent functions for BnAb-conferred protection from in vivo cell free virus challenge was conducted using the b12 BnAb. Since the isolation of b12 numerous anti-HIV-1 BnAbs have been isolated and demonstrated to exhibit enhanced neutralization breadth and potency [1]. The role of Fc-dependent functions in the protective efficacy of some of these BnAbs has been evaluated in mouse models of HIV-1 entry [20]. Inhibition of HIV-1 entry can be assessed in luciferase reporter mice by infusing adenovirus encoding HIV-1 receptor and co-receptor (i.e. CD4 and CCR5), infusing anti-HIV-1 or control antibodies, challenging with HIV-1 pseudovirus and full-body imaging. Employing this system Bournozos et al. [20] demonstrated that BnAbs with murine IgG2a Fc, which confers preferential binding to activating FcγRs, were better able to inhibit HIV-1 entry than BnAbs expressing wild type murine IgG1 or an IgG1 variant with diminished binding to FcγR. Reliance on Fc-dependent functions for optimal in vivo efficacy was observed for a panel of antibodies, suggesting Fc-dependent functions are important independent of the viral epitope targeted by the antibody. That this observation reflected FcγR engagement by antibodies is suggested by the absence of differences in efficacy between antibody variants in mice lacking FcγR expression. Lastly, it is important to note that the authors modified their mouse model by engineering mice to express human FcγRs. This allowed an assessment of the role of Fc-dependent functions for BnAbs expressing human Fc in preventing viral entry. Employing wild type BnAb, as well as versions engineered to exhibit deficient or enhanced FcγR binding, the authors observed evidence for a role for human Fc/FcγR interactions in inhibition of in vivo viral entry. Cumulatively, these observations are largely supportive of the role for Fc/FcγR interactions in BnAb-conferred protection observed in macaques. The results from the murine experiments suggest that the role for Fc-dependent anti-viral functions of BnAbs for optimal protection extends beyond the b12 BnAb and might be a generalizable phenomenon.

Lastly, it should be noted that in addition to triggering anti-viral effector functions, interactions between BnAbs and FcRs are important for sustaining antibody concentrations and antibody transport. Indeed, antibody binding to the FcRn is important for homeostasis and antibody transport to mucosal surfaces. As such, the VRC01 antibody was recently mutated to enhance binding to the FcRn [31]. The resulting VRC01-LS antibody exhibited increased in vitro transcytosis, a 2.5-fold longer in vivo serum half-life in rhesus macaques and a tendency to accumulate in macaque rectal tissues through FcRn binding. Lastly, suboptimal concentrations of VRC01-LS provided enhanced protection against rectal SHIV_BaLP4_ challenge, as compared to VRC01 WT (i.e. 7/12 vs. 2/12 animals protected from infection). These results highlight that Fc/FcR interactions are an important determinant of BnAb-conferred protection, even if the interaction does not directly stimulate anti-viral functions.

## Utility of BnAbs for control of cell–cell virus transmission in vitro

Much evidence highlights the potential utility of BnAbs for preventing HIV-1 infection. A potential caveat for utilizing BnAbs to prevent HIV-1 infection is the presence of cell-associated virus within infectious bodily fluids, such as semen [37]. Cell-associated virus has long been proposed as a mechanism of transmission of HIV-1—the so-called “Trojan Horse” hypothesis [38, 39]. In macaques, cell-associated SIV is highly efficient at initiating infection, more so than cell free virus [40]. In humans, there are limited data based on virus sequencing that cell-associated virus may initiate a proportion of HIV-1 infections [41].

A key component of the “Trojan horse” hypothesis is that cell-associated virus may evade anti-viral immunity [38, 39]. There has been much research into this possibility, particularly with regards to cell-associated virus evading BnAbs. While some publications have reported BnAbs to prevent in vitro cell-to-cell transmission of HIV-1, others have demonstrated a decreased efficacy of BnAbs against cell-associated virus compared to cell free virus [42–51]. These divergent results likely reflect the utilization of different in vitro experimental systems. Nevertheless, decreased efficacy a BnAbs against cell-associated virus has been reported in terms of both higher 50% inhibitory concentrations (IC_50_) and incomplete neutralization [45]. Importantly, the ability of BnAbs to prevent cell-to-cell spread is dependent on the virus/antibody combination [47]. Furthermore, combinations of BnAbs may be more efficient than single antibodies [44].

## BnAb control of cell-associated challenge in vivo

We recently developed a cell-associated SHIV_SF162P3_ infection model in pigtail macaques [52]. The model was adapted from a previously published cell-associated SIV model that used splenocytes from infected macaque donors to initiate infection [53]. Passive transfer of the BnAb PGT121 protected 6/6 pigtail macaques from an intravenous cell free SHIV_SF162P3_ challenge but only 3/6 macaques from an intravenous cell-associated SHIV_SF162P3_ challenge. However, the lack of efficacy in two macaques challenged with the cell-associated SHIV_SF162P3_ appeared due to low levels of BnAb administered. Interestingly, one macaque had no viremia until eight weeks post challenge with cell-associated SHIV_SF162P3_. It appeared that the SHIV_SF162P3_ lay dormant, possibly existing as cell-associated virus in tissues, and only emerged when the passively transferred BnAb waned to low levels. Whether this anecdote will represent a common mode of evading strategies to control HIV-1 with BnAbs is unknown. We recently suggested that, given the capacity of HIV-1 to remain latent under ART for decades, HIV-1 could remain suppressed by BnAbs for years until the antibodies (whether delivered passively or induced by vaccination) wane to sub therapeutic levels [54]. This has implications for the long-term follow up of BnAb based clinical trials.

## The use of BnAbs for HIV-1 therapy and cure: role of Fc-dependent responses

Following the isolation of first generation BnAbs there was much interest in their potential for therapeutic utilization. In an early study in hu-PBL-SCID mice, Poignard et al. observed limited utility of first generation BnAbs in mice infected with HIV-1_JR-CSF_ or HIV-1_SF162_ [55]. Indeed, monotherapy with the CD4 binding site BnAb, b12, did not significantly decrease plasma viral loads in mice infected with either virus, as compared to control animals. Furthermore, several viral isolates derived from animals treated with b12 developed resistance to the BnAb. Similarly, treatment of mice infected with HIV-1_JR-CSF_ with a cocktail of BnAbs (i.e. b12, 2G12 and 2F5) achieved unsatisfactory results. Temporary decreases in viral load were noted, but were followed by viral rebound and escape from one or all three BnAbs.

Trials of first generation BnAbs as therapeutics in HIV-1-infected humans also revealed transient therapeutic benefits followed by viral escape from BnAb. Trkola et al. administered a BnAb cocktail (i.e. 2G12, 2F5 and 4E10) to eight individuals with chronic HIV-1 infection and six individuals with acute HIV-1 infection one day before cessation of ART [56]. Trial participants received 13 BnAb injections over 11 weeks. Two of eight chronically infected donors exhibited a delay in viral rebound, as compared to historical data of the same individuals undergoing a treatment interruption in the absence of BnAb therapy. The acutely infected BnAb-treated participants exhibited a significant time delay prior to viral rebound as compared to a control group of acutely infected individuals undergoing treatment interruption in the absence of BnAb treatment (median 8 weeks vs. 3.75 weeks). The therapeutic benefits of the BnAb cocktail appeared to be primarily driven by 2G12, as viral rebound was accompanied by resistance to 2G12 in 12/14 trial participants. Mehandru et al. reported similar results in another trial assessing the therapeutic potential of the BnAb cocktail of 2G12, 2F5 and 4E10 [57]. As observed by Trkola et al. [56], these investigators noted that BnAb therapy slowed viral rebound following treatment interruption, as compared to historical controls. Furthermore, loss of viral control was associated with resistance to 2G12.

Since these initial trials, numerous BnAbs, with increased potency and breadth compared to first generation BnAbs, have been isolated from HIV-1-infected individuals [1]. The isolation of next generation BnAbs has reinvigorated interest in utilizing BnAbs as therapeutics for HIV-1 infection. Assessment of the therapeutic potential of next generation BnAbs in humanized mouse models suggested monotherapy to be inefficient, transiently controlling viremia before the development of viral resistance to BnAbs [58]. Combination therapy with five BnAbs, however, controlled viremia and did not result in viral resistance to BnAbs. Another study demonstrated that single BnAbs were sufficient to control viremia in a proportion of humanized mice, if viral replication was first controlled by ART and BnAb administrations were initiated prior to cessation of ART [59]. Studies in non-human primates have revealed that both combination therapy and monotherapy with BnAbs can control SHIV replication, but monotherapy can lead to the development of viral escape mutants [2, 11]. Furthermore, therapeutic administration of BnAbs to macaques during acute SHIV infection might facilitate the development of autologous antiviral immunity, thus conferring prolonged control of viral replication [9].

In addition to animal studies, next generation BnAbs have also been screened as therapeutics in HIV-1-infected humans [3, 4, 60]. Monotherapy with 3BNC117 and 10-1074 results in transient control of viremia, and 3BNC117 can delay viral rebound following treatment interruption. As noted in animal studies, viral resistance to BnAbs can develop in humans undergoing monotherapy.

As well as data highlighting the capacity of BnAbs to control viremia, several studies using murine models, as well as modelling of data from a human clinical trial, suggest a role for FcγRs in the therapeutic benefits conferred by BnAbs. A role for BnAb interactions with FcγR for controlling viremia in infected animals was demonstrated by Bournazos et al. [20]. Humanized mice infected with HIV-1 were treated with a cocktail of BnAbs (i.e. 3BNC117, PG16 and 10-1074) modified either to not interact with FcγR (FcR^null^) or to exhibit enhanced binding to activating FcγR. A quicker and sustained control of viremia was observed in animals treated with the antibody cocktail containing antibodies designed to more strongly interact with activating FcγR. Halper-Stromberg et al. demonstrated that treatment of HIV-1-infected humanized mice with a BnAb cocktail (i.e. 10-1074, PG16 and 3BNC117) 4 days post infection reduced the establishment of a latent reservoir, as exhibited by a lack of viral rebound in a proportion of animals after waning of therapeutic BnAbs [61]. A rendition of this experiment using FcR^null^ versions of the BnAbs suggested a role for FcγR interactions in the observed interference with the establishment of a latent reservoir. Significantly more mice had rebounded viremia by 44 days post treatment with FcR^null^ versions of the BnAbs than those treated with wild type BnAbs capable of interacting with FcγR. Lastly, Lu et al. assessed the rate of viral load decline in HIV-1-infected humans treated with 3BNC117 [62]. Modelling suggested that the rate of decline in viremia was too rapid to be explained by neutralization of free virus alone. Indeed, the analysis suggested that non-neutralizing antibody effector functions, such as those involved in eliminating infected cells, were involved in the therapeutic benefits conferred by the antibody. Furthermore, the ability of BnAbs to eliminate human cells infected with the HIV-1_YU2_ laboratory strain or isolates derived from HIV-1-infected patients was demonstrated in vivo in mice. The elimination of infected cells was demonstrated to be dependent on FcγR-mediated recognition of antibody, as infected cells were not eliminated by FcR^null^ BnAbs and in vivo blocking of FcγRs prevented the elimination of infected cells.

## Diversity of Effector cells, FcγRs and antibody isotypes: potential influence on BnAb efficacy

While the BnAb paratope dictates neutralization breadth and potency, accumulating evidence indicates that Fc-dependent functions might be required for BnAbs to optimally protect from infection, suppress viral load and/or clear infected cells [5, 20, 61, 62]. These Fc-dependent functions might include ADCC, ADP, complement activation, effector cell release of cytokines, chemokines or enzymes, inhibition of transcytosis and mucus trapping. An antibody’s Fc functional capacity can be modulated through multiple small biophysical differences of the Fc region [63], including the isotype (e.g., IgG, IgA, IgM, IgE, IgD), subclass (e.g., IgG1-4, IgA1, IgA2) [64], allotype [65, 66] and glycosylation of the Fc heavy chain [67, 68].

In humans, there are three distinct classes of Fcγ receptors: FcγRI, FcγRII (FcγRIIa, FcγRIIb, and FcγRIIc), and FcγRIII (FcγRIIIa and FcγRIIIb), which bind to different IgG subclasses with varying affinity, and can cause either activation or inhibition of the effector cell [64]. These FcγRs are expressed on a wide variety of innate immune cells including NK cells, monocytes, macrophages, dendritic cells, eosinophils, basophils and neutrophils. NK cells almost exclusively express the activating FcγRIIIa and are the effector cells most commonly associated with ADCC [69]. Macrophages, neutrophils, eosinophils, basophils and dendritic cells all express a more diverse range of FcγRs on their surfaces (both activating and inhibitory) and can mediate various effector functions, including ADCC, phagocytosis and trogocytosis (i.e., the exchange of cellular membrane fragments between effector and target cells) [70–72].

Human FcγRs are diverse. A range of FcγR polymorphisms have been identified, some of which have greater Fc binding affinity and are associated with enhanced Fc effector function capacity. For example, FcγRIIa has two common polymorphisms—H131 and R131. The FcγRIIa H131 polymorphism, which is commonly associated with enhanced ADP, is also related to HIV-1 disease progression status [73, 74]. The FcγRIIIa is also known to exhibit polymorphisms at position 158–V158 and F158. The high affinity FcγRIIIa V158 polymorphism, is associated with enhanced ADCC functionality and with better outcomes for cancer monoclonal therapeutics [75, 76]. Surprisingly, the FcγRIIIa V158 polymorphism might associate with HIV-1 disease progression [77] and associates with the risk of infection in recipients of the VAX004 vaccine [78].

While many factors can contribute to the Fc-dependent functions of antibodies, several studies have generated HIV-1-specific BnAbs with modified capacities to bind FcγRs and mediate Fc-dependent functions [5, 25, 36]. The aim of such studies is to gain an understanding of how to improve BnAb-conferred protection from infection. While abrogation of the Fc-dependent activity of BnAb b12 through introduction of the LALA mutation decreased the protective efficacy of the BnAb [5], a non-fucosylated version of b12, which exhibited an enhanced ability to bind to FcγRIIIa and trigger ADCC, did not confer any additional protection from repeated low-dose viral challenge [36]. These data suggest that engagement of FcγRIIIa and the triggering of ADCC activity are likely not essential to achieve in vivo BnAb-conferred protection from viral challenge. Alternatively, BnAbs that trigger a wide range of Fc-dependent functions or ‘polyfunctional’ Fc activity may be optimal. Indeed, several studies suggest that polyfunctional non-neutralizing Fc-dependent functions of HIV-1 binding antibodies can contribute to enhanced viral control and protection from infection [79–82]. Furthermore, the presence of antibodies with Fc polyfunctionality may contribute to the development of BnAbs. Richardson et al. recently observed that individuals that develop BnAbs have higher Fc polyfunctionality and increased subclass diversity [83].

It is important to note that studies have also assessed the effect of modifying the isotypes of BnAbs, especially to IgA, with varying results [84–88]. Isotype switching of 2F5 from IgG1 to IgA2 improved epitope affinity and improved inhibition of HIV-1 transcytosis [84]. In contrast, other studies have reported 2F5 IgA or 2F5 IgM variants to provide inferior protection against HIV-1 compared to 2F5 IgG [85, 86]. Indeed, 2F5 IgM failed to inhibit HIV-1 transcytosis [85], while 2F5 IgA failed to neutralize HIV-1 infection of PBMC [86]. In contrast, 2F5 IgG protected against HIV-1 infection in vitro and intravenous administration of 2F5 IgG protected macaques from intravaginal viral challenge [86]. Importantly, intravenous administration of 2F5 Fab exhibited no protection, emphasizing the importance of the Fc region of the 2F5 BnAb for protection. An additional study modified the V3 neutralizing antibody HGN194 from IgG1 to dimeric IgA1 and dimeric IgA2. The resulting antibodies exhibited similar neutralization potencies, but HGN194 dimeric IgA1 provided the best protection in vivo against intrarectal viral challenge. Protection was correlated with in vitro measurements of inhibition of viral transcytosis and virion capture [87]. Lastly, neutralizing CH31 IgG antibodies exhibited enhanced protection against intrarectal viral challenge in macaques compared to monomeric, dimeric or secretory IgA2 variants [88]. Clearly, these studies indicate that the isotype of BnAbs can contribute significantly to protection from mucosal viral challenge, but the contribution of isotype might be epitope specific and too few studies have been conducted to determine how the antibody isotype contributes mechanistically to protection. Similar to IgG, IgA can engage its Fcα receptor that is present on the surfaces of monocytes, macrophages and neutrophils to mediate phagocytosis, respiratory burst, and the release of various cytokines and inflammatory mediators [89]. The potential of Fc/FcR interactions between IgA and Fcα receptor is an area of research that has not yet been fully explored in terms of its potential for combatting HIV -1.

## Potential importance of diverse human NK cell functionality on BnAb efficacy

In addition to polymorphisms in FcγRs, several additional variables might potentially impact the ability of effector cells to utilize BnAbs to mediate Fc-dependent functions. NK cells and the impact of the processes of education and differentiation on their functional potential best represent this.

Diversity in the capacity of NK cells to respond to stimuli is introduced through the process of NK cell education [90]. During the education process NK cells scan the self-environment for constitutively expressed ligands to their activating and inhibitory receptors. The receptors involved in this process include activating and inhibitory killer immunoglobulin-like receptors (KIR), which recognize classical major histocompatibility complex class I (MHC-I or HLA-I) molecules [91], and the inhibitory NKG2A receptor, which recognizes the non-classical HLA-E [92]. In general, education tunes the potential responsiveness of an NK cell in a manner that maintains self-tolerance, conferring functional capacity to cells expressing inhibitory receptors that recognize self-ligands [93–95] and reducing the functional capacity of cells expressing activating receptors that recognize self-ligands [96]. Several studies have now demonstrated that education determines the responsiveness of NK cells to direct stimulation with HLA-I-devoid target cells [93, 94], as well as FcγR-dependent stimulation of NK cells with antibody-coated target cells [93, 95].

The role of NK cell education in determining Fc-dependent NK cell functions via anti-HIV-1 antibodies has been investigated using HIV-1 envelope coated target cells and polyclonal antibodies derived from patients [97–100]. Most of these studies have evaluated the role of education in anti-HIV-1 Fc-dependent NK cell responses by focusing on single education-competent receptor/ligand combinations, such as KIR3DL1/HLA-Bw4 or KIR2DL1/HLA-C2. The highlighted studies have pointed to higher levels of antibody-dependent activation in NK cells educated through the studied receptor than in an autologous NK cell population containing both non-educated cells and cells educated through other inhibitory receptors. Isitman et al. compared anti-HIV-1 ADCC mediated by PBMC from individuals with NK cells educated through KIR3DL1/HLA-Bw4 and individuals with NK cells lacking education through KIR3DL1/HLA-Bw4 [101]. No differences in ADCC were noted between the two groups, leading the investigators to suggest that NK cell education may not be important for determining anti-HIV-1 ADCC capacity. Alternatively, it is possible that NK cells educated through inhibitory receptor/ligand combinations other than KIR3DL1/HLA-Bw4 conferred compensatory NK cell education in the individuals lacking this receptor/ligand combination. We recently attempted to evaluate the relative contributions of educated and non-educated NK cells within PBMC to anti-HIV-1 ADCC [99]. Briefly, PBMC were stained with fluorochrome-conjugated antibodies to identify cells expressing inhibitory receptors that would educate NK cells, given the donor’s HLA-I profile. Stained cells were FACs sorted as a population enriched for educated NK cells (i.e. education^+^ PBMC) and unstained cells were sorted as a population lacking educated NK cells (i.e. education^−^ PBMC). Utilizing cell numbers reflecting the frequency of each population of cells within total PBMC, we evaluated the relative ability of each cell population to kill gp120-coated target cells in the presence of anti-HIV-1 antibodies. We observed robust ADCC mediated by total PBMC that was recaptured by the sorted education^+^ PBMC. In contrast, the sorted education^−^ PBMC mediated little-to-no ADCC, and were significantly less cytotoxic than either the whole PBMC or the education^+^ PBMC population.

While our data imply that educated NK cells are the primary mediators of anti-HIV-1 ADCC within PBMC, it is important to note that these data were collected using gp120-coated target cells and with polyclonal antibody mixtures. Future experiments are needed to determine if education has a similar impact on the capacity of NK cells to utilize BnAbs to mediate anti-HIV-1 ADCC. Furthermore, it would be ideal to conduct these experiments with autologous HIV-1-infected target cells, which present viral envelope in physiological conformations and amounts. The implementation of autologous infected cells will also address the role of autologous HLA-I in inhibiting anti-HIV-1 ADCC, and how downregulation of HLA-I by HIV-1 nef influences the ability of self-HLA-I to inhibit anti-HIV-1 ADCC [102].

In addition to education, the relative responsiveness of an NK cell to antibody-dependent stimulation is determined by the stage of differentiation of the cell. NK cells differentiate in a defined pattern, proceeding from CD56^bright^ to CD56^dim^ before gaining expression of the CD57 differentiation marker [103]. Throughout this process NK cells gain expression of KIRs and FcγRIIIa and become more cytotoxic. Additionally, differentiated CD56^dim^CD57^+^ NK cells exhibit more robust responses through FcγRIIIa following stimulation with anti-receptor antibody or anti-viral antibody-coated target cells expressing HIV-1 antigens [100, 104].

Importantly, viral infections appear to influence the NK cell differentiation process. Indeed, individuals infected with human cytomegalovirus (HCMV) exhibit expansions of differentiated CD56^dim^CD57^+^ NK cells that also express the activating NKG2C receptor, which recognizes the non-classical HLA-E molecule [92, 105]. These NK cells exhibit robust function through FcγRIIIa [106]. Furthermore, these differentiated NK cells degranulate following ligation of NKG2C [105]. Interestingly, CD56^dim^CD57^+^NKG2C^+^ NK cells have been noted to occur in HIV-1-infected individuals in a HCMV-dependent manner [107]. The frequency of these cells in HIV-1-infected donors, however, appears to be exaggerated compared to HCMV-infected HIV-1-uninfected donors. Further research is required to determine the anti-HIV-1 antibody-dependent functions of CD56^dim^CD57^+^NKG2C^+^ NK cells. It will be interesting to determine if these cells can confer enhanced anti-viral benefits in individuals receiving BnAbs for the purposes of therapy or cure.

Lastly, the ability of NK cells to mediate Fc-dependent functions is influenced by virus-induced alterations on target cells. Much has been published on the ability of HIV-1 nef and vpu to downregulate CD4 on infected cells, prevent exposure of CD4-induced epitopes on viral envelope and facilitate the evasion of ADCC mediated by polyclonal patient-derived ADCC antibodies that predominantly recognize CD4-induced envelope epitopes [108, 109]. This phenomenon is less of a problem for BnAb-mediated ADCC, as BnAbs tend to recognize HIV-1 envelope in its native CD4-unbound trimeric state. In addition to downregulating CD4, HIV-1 nef can downregulate the expression of ligands for the activating NKG2D NK cell receptor, which can serve as a co-receptor for anti-HIV-1 ADCC [110–112]. Furthermore, HIV-1 vpu can downregulate cellular tetherin, which plays an essential role is concentrating virus at the cellular membrane. Downregulation of tetherin decreases the amount of viral antigen available on the surface of infected cells and is a means of evading anti-HIV-1 ADCC [113]. The potential for HIV-1 accessory proteins to influence ADCC readouts highlights the importance of carefully selecting viruses and assays that most closely portray the in vivo situation in which the antibody in question will be immersed [112, 114].

## Conclusions

The weight of data supports the contention that Fc-mediated functions of BnAbs are important to their efficacy in preventing HIV-1 and controlling viremia. This contribution is likely to be even more important in the context of exposure to cell-associated virus, where virus may evade neutralization by BnAbs. Newer generation BnAbs have higher potency and are potential tools for preventing and controlling HIV-1 infection. Additional work characterizing the in vivo importance of Fc-mediated functions of newer generation BnAbs is needed.
